# Developing community-based surveillance systems for vaccine-preventable diseases: lessons learned from Indonesia

**DOI:** 10.1080/16549716.2025.2548083

**Published:** 2025-08-28

**Authors:** Galuh Sitorukmi, Cici Permata Rusadi, Lutfan Lazuardi, Sabarinah Prasetyo, Likke Prawidya Putri

**Affiliations:** a Center For Health Policy and Management Faculty of Medicine, Public Health and Nursing Universitas Gadjah Mada, Yogyakarta, Indonesia; b Department of Health Policy and Management Faculty of Medicine, Public Health and Nursing Universitas Gadjah Mada, Yogyakarta, Indonesia; c Department of Epidemiology, Faculty of Public Health, Universitas Indonesia, Depok, Indonesia

**Keywords:** community-based surveillance, community health volunteers, vaccine-preventable disease, low- and middle-income countries, communicable disease, community health reporting

## Abstract

**Background:**

Routine immunization was severely disrupted during the COVID-19 pandemic, leading to a resurgence of vaccine-preventable diseases (VPDs) in Indonesia. While community-based surveillance (CBS) was instrumental in COVID-19 detection, its role in VPD monitoring remains limited.

**Objective:**

This study explores practices, challenges, and future opportunities to develop a CBS for vaccine-preventable diseases (VPDs).

**Methods:**

We applied a convergent mixed-method study in Sleman regency, Indonesia. We interviewed 41 stakeholders – health staff, community health volunteers (CHVs), and community leaders – and surveyed 70 caregivers of under-5. Interviews were analysed using content analysis according to the health systems combined with public health surveillance frameworks. The survey was examined descriptively.

**Results:**

Existing community involvement in communicable disease surveillance includes systematized reporting for TB, dengue, and COVID-19 using tools like mobile apps and formal guidelines, and non-systematized case reporting for leptospirosis and VPDs. While health facilities have established procedures for recording, reporting, and responding to VPDs, community roles remain undefined. Challenges include CHVs’ limited surveillance knowledge, high workloads, lack of digital literacy, inconsistent incentives, absence of case definitions and reporting procedures, and resource shortages. Additionally, unintegrated reporting systems hinder health staff from utilizing and monitoring CBS reports effectively. Future CBS development should focus on neighbourhood committees and CHVs, with 61% of surveyed community members willing to participate.

**Conclusion:**

Strengthening CBS for VPDs requires formalized guidelines on community-to-health facility reporting, integration of both conventional and digital reporting systems, improved coordination with facility-level systems, certified CHV training, and a supportive policy and financing framework to ensure long-term sustainability.

## Paper Context

- **Main findings:** In Indonesia, while guidelines of recording and reporting vaccine-preventable diseases from the health facilities to the wider surveillance systems has long been established, no such formal procedures of reporting from the community to health facilities; this is one of the challenges that caused late detection of the vaccine-preventable diseases.
- **Added knowledge:** This article adds to the body of knowledge on technical aspects that are critical to preparing for community-based surveillance for infectious diseases, particularly vaccine-preventable diseases, namely the existence of simple case definitions and reporting guidelines that can be adapted to electronic or paper-based disease reporting systems.
- **Global health impact for policy and action:** given the growing importance of community involvement in health, mapping and compiling the various terminologies, strengths and interests of community members can be useful in identifying community members who are important to engage deeply in delivering population health interventions

## Background

The coronavirus disease 2019 (COVID-19) pandemic has reiterated the importance of engaging communities to achieve health system resilience. Prior to the pandemic, community-based surveillance (CBS) was widely implemented to early detect communicable diseases, such as Ebola, tuberculosis (TB), measles, and polio, and symptoms of infectious diseases and to monitor health outcomes [1–7]. During the pandemic, community members further improved preparedness by supporting health professionals in contact tracing and testing and executing rapid response efforts [8–12]. Thus, community members, including community health volunteers (CHVs), community leaders, religious leaders, and women’s groups, have contributed to minimizing the health impact of the pandemic [13–16].

CBS is a vital element for effective disease detection and response, leveraging community members to report public health information and enabling early outbreak detection and rapid control measures [17–19]. The integration of CBS into formal surveillance systems supports early warning, verification, and response capabilities and addresses gaps in facility-based systems, especially in developing countries and crisis settings [1,17]. CBS also strengthens the district-level capacity to detect, verify, and investigate health-related events while fostering resilient community networks and increasing public awareness of infectious diseases [18,20]. In low- and middle-income countries (LMICs), where healthcare infrastructure may be stretched thin, early detection by community members can provide a critical window for intervention, preventing the spread of infectious diseases [21].

In Indonesia, an LMIC with a decentralized health system, a range of initiatives have demonstrated community involvement in surveillance. The Indonesian Red Cross piloted a Community Epidemic and Pandemic Preparedness program in 16 villages, achieving high predictive accuracy for diseases, such as dengue, COVID-19, TB, and leptospirosis [22]. The *Puspa* program, introduced during the COVID-19 pandemic, trained CHVs to map high-risk populations and monitor infected individuals [23]. A long-standing dengue control program, the *Jumantik* program, engages community volunteers in mosquito vector monitoring and breeding prevention [24,25].

Indonesia’s 2014 Health Surveillance Regulation recognizes the community’s role in reporting cases, offering resources, and supporting data management [26]. A 2024 technical guide outlines CBS implementation for local leaders, but inconsistent subnational regulations may hinder community participation in the country’s decentralized governance system [27].

The World Health Organization (WHO) announced the end of the COVID-19 global health emergency in May 2023, but with the warning that COVID-19 remains a global threat [28]. Studies have reported the negative impact of the pandemic on essential health services, including routine vaccination of children [29,30], which can lead to an increased morbidity from vaccine-preventable diseases (VPDs) [31]. In 2022, in the Sleman Regency, three outbreaks related to VPD occurred: one case of measles, one case of pertussis, and two cases of rubella [32]. Furthermore, in 2024, an outbreak of acute flaccid paralysis occurred in Central and Eastern Java, requiring Sleman to conduct mass polio vaccination [33]. In late 2023, suspected cases of Japanese encephalitis, a zoonotic VPD, were detected in Sleman, although follow-up laboratory testing ruled it out. Nonetheless, the episode prompted Sleman’s designation as an endemic area and triggered a mass vaccination campaign in the following year [34].

With the increasing trend in VPDs and the potential for newly emerging diseases, it is critical to develop a model for the CBS of communicable diseases and their risk factors. Thus, in this study, we aimed to explore the practices, challenges, and future opportunities of developing a CBS program for infectious diseases in Indonesia, focusing on VPDs.

## Methods

### Overview

This study is part of a research consortium on CBS in three areas of Indonesia. We applied mixed methods by conducting the following: (1) a qualitative study using semi-structured interviews with health authorities, health workers in public community health centres (*Puskesmas*) and hospitals, CHVs, village and sub-district leaders, and key community members, and (2) a quantitative study using a survey of mothers with children aged < 5 years. Because of the differing elements and approaches required for the CBS of zoonotic versus non-zoonotic VPDs, this study focused on non-zoonotic VPDs included in Indonesia’s routine immunization schedule, consistent with the current Indonesian health policy.

### Participant selection

Our study covers Sleman Regency, a district located in Yogyakarta Province, Java Island, the most populated island in Indonesia, with more than half of the population residing in urban areas. Sleman Regency has approximately 1.2 million inhabitants and an area of 574.8 km^2^.

#### Interviews

The interviews explored current practices and challenges of community involvement in disease surveillance. Two sub-districts were selected based on discussions with key stakeholders (i.e. head of the directorate of communicable disease, Sleman Health Authority, and their team) and known contact with the authors. Participants from each sub-district included staff from *Puskesmas* (health workers responsible for surveillance and communicable diseases), sub-district authority offices, and village authority offices. In each *Puskesmas*, we invited health workers in charge of surveillance and/or communicable diseases and CHVs (*kader*). We invited the staff in charge of health matters in each sub-district and the village authority office. Two academic-affiliated referral hospitals for VPDs were also included, irrespective of geographic location, as they were the main referral facilities for VPDs (Supplementary Figure A).

The respondents were selected using purposive sampling. Inclusion criteria required involvement in infectious disease surveillance for at least 6 months during the COVID-19 pandemic between March 2020 and June 2022, responsibility for COVID-19 surveillance (testing and tracing), and working within the selected sub-district. Individuals who were unable to communicate with Indonesians were also excluded.

#### Surveys

This survey examined current practices and future opportunities for developing CBS for infectious diseases, particularly VPDs. Participants included mothers or caregivers of children aged < 5 years attending *Posyandu* sessions and community-based health activities conducted by volunteers under *Puskesmas* supervision. The research team obtained *Posyandu* schedules from *Puskesmas* and randomly selected sessions to visit. Thirty-five mothers or caregivers were included from each of the two sub-districts.

### Instrument development

The interview guides and questionnaires were collaboratively developed by the research consortium. The interview guide combines the WHO health systems building blocks [35]; a health systems resilience framework that explores the aspects of governance, financing, service delivery, human resources, medical technology, and health workforce with the community at the centre [13]; and McNabb et al.’s public health surveillance framework, outlining functions, such as detection, reporting, analysis, and response [36].

The questionnaire on the knowledge of and attitude towards COVID-19 and VPDs was adapted from previous studies [37–39]. Both instruments were piloted with health staff and CHVs in another district in Yogyakarta Province.

#### Data collection

The survey was administered between December 2022 and January 2023, whereas interviews took place from November 2022 to July 2023. Group interviews were conducted with CHVs and *Puskesmas* health professionals, and individual interviews were conducted with staff from health authorities, hospitals, sub-district offices, and village governments. All interviews were conducted in the participants’ workplaces, whereas surveys were completed either in the respondents’ homes or at the *Posyandu* sites. Three female researchers, GS, CR, and LP, each holding a minimum Master of Public Health degree, conducted the interviews. Two trained enumerators with bachelors’ degrees in health sciences and prior survey experience administered this survey. None of the research team members had a preexisting relationship with the participants. Written informed consent was obtained from all the respondents before data collection commenced.

#### Data analysis and trustworthiness

Survey data collected on paper were entered into the KoboToolbox and descriptively analysed using Stata/MP 17.0 or Excel. The interviews were transcribed and deductively coded based on a predeveloped framework, with additional codes created for emergent themes. The anonymised findings were triangulated through discussion sessions with respondents and stakeholders to ensure clarity and trustworthiness.

## Results

Forty-one health authority staff, district hospital staff, *Puskesmas* staff, CHVs (*kader*), sub-district government staff, and village government staff members were interviewed. None of the participants refused participation. This survey involved 70 mothers or caregivers who attended monthly community-based health programs where their children aged < 5 years were monitored for their growth and development (the *Posyandu* program); all were women. Three caregivers declined to participate owing to personal reasons. Details of the respondents’ characteristics are presented in Table 1.

**Table 1.** Respondent characteristics.

Characteristics	Description	Total (N = 41)	Group/individual interview
**Qualitative interviews**			
Sex	Male	12 (29%)	0/12
	Female	29 (71%)	4/25
Institution	District health authority	4 (10%)	0/4
	District hospital	8 (20%)	0/8
	*Puskesmas*	9 (22%)	4/5
	Community health volunteers (*kader*)	4 (10%)	4/0
	Sub-district government	8 (20%)	0/8
	Village government	8 (20%)	0/8
**Survey**			
Sex	Female	70 (100%)	N/A
Age group	22–29 years	21 (30%)	N/A
	30–39 years	34 (49%)	N/A
	≥40 years	15 (21%)	N/A
Profession	Not employed	47 (67%)	N/A
	Employed*	14 (20%)	N/A
	Self-employed	8 (11%)	N/A
	Laborers	1 (1%)	N/A
Caregiver status	Parents	65 (93%)	N/A
	Others (grandparents, aunts)	5 (7%)	N/A
Educational attainment	Elementary school	2 (2.9%)	N/A
	Junior/senior high school	50 (71.4%)	N/A
	Diploma/bachelor degree	18 (25.7%)	N/A

Notes: *Employed includes civil servant, private employee, and honorary worker; self-employed includes entrepreneur.

## Practice

This section presents the results of the survey of mothers or caregivers and interviews with stakeholders, staff, and CHVs.

### Types of activities involving communities: results from interviews and the survey

The interviews revealed community activities in disease surveillance, including raising awareness, recording and reporting diseases or symptoms, and responses (Table 2). The survey results were aligned, identifying key activities, such as disseminating COVID-19 information (44%), reporting cases via SMS/WhatsApp (30%), and collecting data on suspected cases (28%). COVID-19 case reporting was primarily conducted through text messaging, WhatsApp, and other calls. Reports were made to neighbourhood committees (43%); *Puskesmas* (38%); and CHVs, midwives, or sub-village leaders (38%). Of the five respondents who tracked other infectious diseases, three reported it solely to *Puskesmas*, one to both *Puskesmas* and community leaders, and one to a hospital (Supplementary Table A).

**Table 2.** Brief information on practice, challenges, and future opportunities on community-based surveillance.

Key topics	Themes	Findings
**Source of information: interviews**		
Practice	Activities in which community was involved	Raising awareness: health campaign during the Posyandu* sessions or in WhatsApp groups
		Recording and reporting: detection of houses with mosquito larvae by community health volunteers (CHVs), screening of family members and neighbors of positive TB cases by CHVs, tracing of positive COVID-19 cases by CHVs
		Responses: CHVs distributed larvicides, accompanied patients with suspected TB to undergo further examinations at health facilities, helped register communities for COVID-19 tests
	Model of community involvement	Systematized: usually involved CHVs, with written guidelines or consensus on how to record and report the cases to health authorities (Figure 1)
		Non-systematized: usually involved the community directly reporting the disease to health authorities (see Figure 2)
Challenges	Human resources	Limited number of CHVs with overburdened tasks
		Lack of specific capacity building on disease surveillance among CHVs
		Lack of an appropriate educational background among the health staff
	Information systems and technology	Low proficiency and familiarity in using the electronic reporting and recording systems
		The presence of multiple unintegrated recording and reporting systems for same diseases
	Financing	Varied and inconsistent incentives for CHVs
		Low amount of incentives compared with the expected tasks
		Limited funding availability provided by the health sector or facilities for the responses
	Governance and leadership	Lack of clear procedures and technical guidelines for recording reporting, and responding
		Monitoring and evaluation by the health authorities did not include the participation rate, simplicity and flexibility of the surveillance system
**Source of information: surveys**		
Practice		
Future opportunities:	Actors to be involved	*Rukun tetangga* and *Rukun warga* leaders, local leaders (*dukuh*), all community members, community figures, village heads, community leaders (*pamong* masyarakat), local youth, village midwives
	Proposed activities to be performed	Case reporting, program dissemination, data collection, health education, supporting environmental health, accompanying suspected patients to health facilities

**Figure 1.**
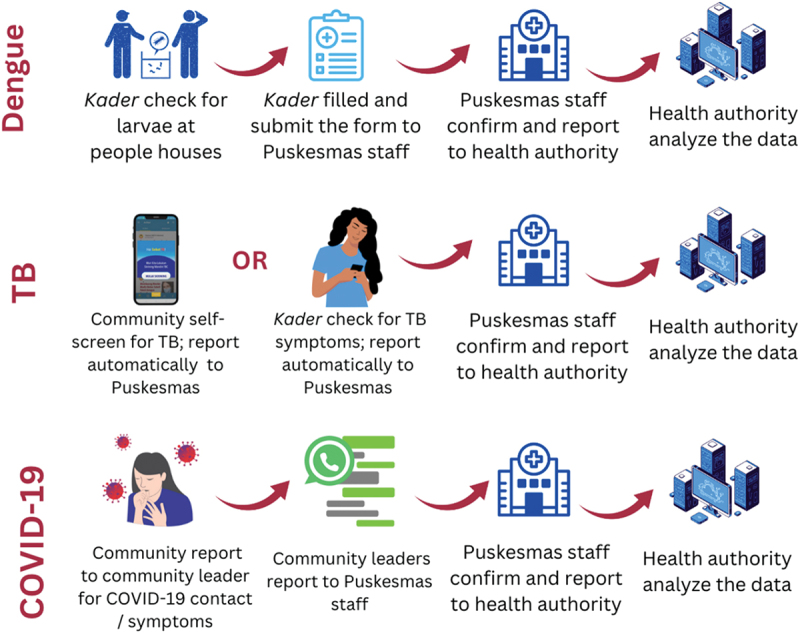
Systematized procedure in infectious disease recording and reporting from community to the health authorities.

Notes: *Kader* = community health volunteers

**Figure 2.**
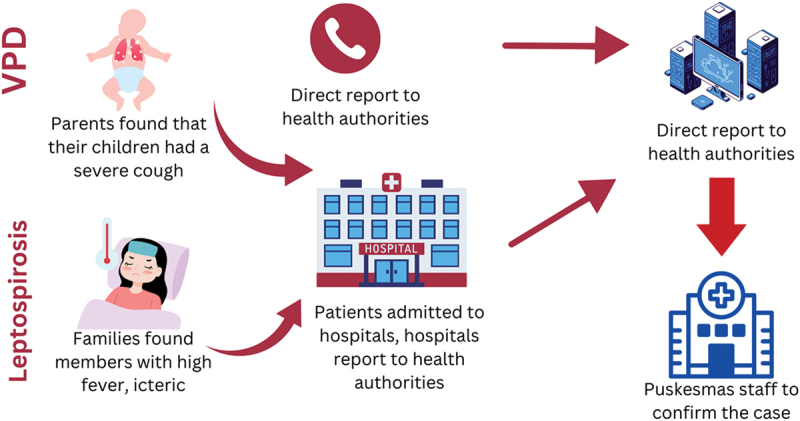
Non-systematized procedure in infectious disease recording and reporting from community to the health authority.

Notes: VPD = vaccine-preventable disease

### Types of infectious diseases that involved the community: results from interviews

Based on the interview results, the CBS activities varied based on disease, and we grouped them into systematized and non-systematized (Figure 1). We considered community participation as ‘systematized’ when meeting either (a) the availability of reporting guidelines OR (b) using a specific form or application for reporting/recording or (c) the availability of hotline disease reporting, as outlined in CBS characteristics by Guerra et al. (2019). Systematized participation was observed for dengue, TB, and COVID-19, whereas non-systematized efforts included diseases such as leptospirosis and VPDs (measles or diphtheria).

In dengue surveillance, CHVs inspected households for mosquito larvae, recorded data on paper, and submitted biweekly reports to the *Puskesmas* staff. For TB, CHVs screened close contacts using a mobile application (e.g. SOBAT TB), which was directly transmitted to the *Puskesmas* staff. CHVs also supported TB treatment adherence through a non-governmental organization (NGO)-supported initiative, providing incentives for case detection, tracing, and investigation. The CBS in COVID-19 surveillance extended beyond CHVs to include community leaders and members (i.e. sub-village heads [*dukuh*], neighborhoods, and hamlet committees [*rukun tetangga/rukun warga*]. Suspected cases were reported via group messaging platforms, such as WhatsApp, where *Puskesmas* staff can directly receive these reports and integrate them into district surveillance systems. Military officers (i.e. *Babinsa/Babinkamtibmas*) supported the taskforce – enforcing social restrictions, regulating event permits, and ensuring that suspected cases sought timely medical care.

Meanwhile, for other diseases, such as leptospirosis, pertussis, and acute flaccid paralysis, the health authorities received notifications from either hospitals or *Puskesmas* staff or directly from the community, without specific procedures on how the community should report the presence of symptoms of probable severe diseases to the health facility; hence, they were considered ‘non-systematized.’ For example, the index case of leptospirosis in 2021 was reported directly to the district health authority by hospital staff, whereas the case of pertussis was reported directly through the family’s acquaintance with the district health authority (Figure 2).

## Challenges

Information on the challenges described in this section was mostly derived from the interviews. When necessary and available, the survey data were used to triangulate the interview findings.

### Human resources

Human resource challenges span all levels, from district health authorities to *Puskesmas* and CHVs, and include staff shortages, heavy workloads, and gaps in knowledge and motivation. District surveillance units are often understaffed, with one or two officers covering multiple programs. Many did not have formal epidemiology training or specialized surveillance courses, forcing them to self-learn and complete their duties. Although some *Puskesmas* staff members receive annual training, limited participation and weak peer-to-peer sharing dilute its impact:

… The vector-related diseases (P2B2) program is held by a single individual at the Health Office. Surveillance staff, environmental health personnel, and leptospirosis programmers may have multiple roles in the field. (Surveillance officer, District Health Office)

CHVs faced similar constraints: few volunteers, heavy workloads, insufficient training, and minimal incentives. Recruitment challenges leave an aging cadre juggling multiple unpaid roles – from environmental health to TB and *Posbindu* (noncommunicable disease [NCD] screening) – while relying on other jobs for income. This contributed to low motivation, posing a challenge for the development of a CBS system that requires active CHVs involvement:

When discussing kader within the community, their responsibilities are already too many… (Puskesmas staff, sub district-B)

The lack of CHV knowledge about surveillance and infectious diseases was attributed to the non-health education background, compounded by the limited capacity building provided. This could have lowered the confidence of the CHVs in promoting health in the community:

…most of these kader do not have a background in health, so talking about diseases is somewhat awkward… I asked one kader during the rapid response training to provide information about Leptospirosis, and when talking about Leptospirosis, it was a bit difficult for them, because they do not have educational background in health, so they are not familiar with health terms, thus their self-confidence is lacking… (Puskesmas staff, sub district-B)

### Information systems and technology

Although the development of information systems and the effective use of technology can speed up the process of recording, reporting, and follow-up surveillance activities, thus encouraging community participation, CHVs face challenges. First, there is a lack of proficiency and familiarity in using applications, which leads to suboptimal use. Some CHVs preferred manual recording and reporting on paper:

…the SOBAT TB application was helpful, because the data in the application is complete. But it is an obstacle for kader who are not familiar with the system or application, so for reports they usually write manually. Then they report it to us, and then we enter it into SITB… (Puskesmas staff, Sub-district B)

Second, surveillance officers faced heavy workloads owing to multiple unintegrated recording and reporting systems. For example, various communicable disease surveillance systems must be recorded on disease-specific platforms mandated by the Ministry of Health (MoH), whereas district health offices also maintain parallel reporting systems, forcing staff to enter the same data multiple times:

…for example, diarrhea has 1 report, ARI (acute respiratory infections) has another, COVID-19 has its own report, then EWARS which also has its own report. So, it is more than double data entry. Actually, we have our own report, the Puskesmas report is called SP2TP while at the same time we have our own reports for each program… (Puskesmas staff, Sub-district B)

### Financing

Although routine specimen tests and other health office tasks are funded, community participation suffers from inconsistent incentives for CHVs. Payments differ based on program and source: village governments, *Puskesmas*, and NGOs, often creating inequality. For example, village governments provide annual incentives ranging from Indonesian Rupiah (IDR) 300,000 to 450,000 (US dollars [USD] 19–28) for CHVs with formal assignment letters, regardless of their performance. *Puskesmas* offer transport fees for COVID-19 tracing or monthly allowances for MCH activities, which are inconsistent across programs. NGOs provide performance-based incentives for TB investigation. Newer programs, such as those for NCDs and mental health, lack incentives. The lack of standardized, performance-linked incentives undermined CHV motivation and program effectiveness, underscoring the need for equitable and standardized support:

Actually, in the past, the kader for the maternal and child health program had received incentives per month … we planned to give (non-financial) incentives like t-shirt … because (the financial incentive) became jealousy between kader. (Puskesmas staff, sub-district A)

Furthermore, the amount of incentives (USD 19–28 annually) was considered low compared with the minimum monthly wage of IDR 2,400,000 (USD 1,560). Some CHVs must cover some of the costs of activities in advance, which is not always reimbursed by the program. This has become one of the hindrances to recruiting new *CHVs*, as minimum financial incentives mean that they cannot recruit *CHVs* with adequate competence, as required by the MoH:

Well … it’s (the task) hard or not, we are ready to go into the community, we are social (workers), we are sincere, we remain enthusiastic. Actually, if the community supports us, the community is happy, we are happy. Even when contribute some money for the activity, it’s something that we are used to… (kader, sub-district A)

However, funding to respond to reported cases remained scarce. Control measures often exceeded district health and *Puskesmas* budgets, requiring greater governmental support. COVID-19 tracing, for example, demanded resources for patient transport, swab logistics, and community isolation – costs that the *Puskesmas* could not cover:

There was no special funding for the surveillance for both task force officers and COVID-19 officers in our village. In the sub-village, there was no such funding, even the formation of the (COVID-19) team was impromptu. But we still receive some transportation cost from Puskesmas. (kader, sub-district B)

### Governance and regulation

The main challenge in involving the community in disease surveillance was the lack of clear procedures and technical guidelines for recording and reporting cases and follow-up responses. Although some diseases, such as TB and dengue, already have mechanisms for collecting reports from the community, there is no specific mechanism on how and what symptoms the community needs to report to healthcare workers, such as VPDs and leptospirosis:

Perhaps we need a standard operating procedure, especially for health matters. In Puskesmas, there was no specific procedure on what to do when we have reports (of infectious disease) from the community. (Puskesmas, staff sub-district A)

Additionally, there is a lack of monitoring and evaluation of the acceptability, simplicity, and flexibility of the surveillance system. For dengue, the *Puskesmas*’ surveillance officer routinely checked the validity of dengue cases reported by residents, but did not monitor acceptability, that is, the participation rate of the community in conducting larval self-examination. Similarly, for TB, the acceptability aspect of mobile application has been assessed by evaluating the participation rate; however, simplicity and flexibility have not been evaluated.

## Future opportunities

### Actors to be involved in community-based surveillance based on respondents’ perspectives

We asked the respondents about the community members they thought should participate in disease surveillance. More than half of the respondents (61%) stated that *rukun tetangga* leaders should be involved, followed by CHVs (58%), and neighborhood associations (*rukun warga*) (41%). Respondents also identified other actors, including village midwives, local leaders (*dukuh*), the entire community, individuals, community figures, village heads, community leaders (*pamong masyarakat*), and youth (Supplementary Table B).

### Willingness to get involved in community-based surveillance

Regarding willingness to participate in disease surveillance, 61.4% of the respondents wanted to be involved in CBS activities. The proportion of those who wanted to be involved was higher among respondents aged ≥40 years (73.3%) and 22–29 years (61.9%) than among those aged 30–39 years (55.9%) (Supplementary Figure B).

Regarding the types of activities that the respondents were willing to perform, 20.9% were willing to perform case reporting and 20.9% program dissemination. Fewer respondents were required to collect data (16.3%), provide health education (11.6%), support environmental health (11.6%), or report suspected cases to health facilities (7%) (Supplementary Table C).

## Discussion

This study identified various opportunities for community involvement in communicable disease surveillance in Sleman. Systematized methods exist for reporting of dengue larvae, TB, and COVID-19 cases; however, no standardized procedures exist for diseases such as pertussis, diphtheria, acute flaccid paralysis, and leptospirosis. Although approximately 40% of the community members participated in health promotion during the COVID-19 pandemic, fewer reported infectious diseases in health facilities. Challenges in recording and reporting included limited knowledge, inadequate training, low motivation, task overload among CHVs, insufficient incentives, and absence of procedures or guidelines. Although several mobile applications are available, many CHVs are unfamiliar with these applications. Health workers also face challenges owing to numerous fragmented electronic disease reporting systems, which overwhelm them in performing routine disease reporting and monitoring tasks.

The debate on whether community health workers or volunteers (CHWVs) should have accredited trainings and formal recruitment processes and remuneration, versus on a voluntary basis, continues. CHVs often lack essential knowledge of key health topics, despite their broader community role relative to formal health workers [40,41]. National policy outlines 25 core competencies and tiered responsibilities for CHVs; however, training remains unstructured, unrecognized as a professional qualification, and does not address the contextual needs [42]. The WHO recommends providing adequate formal training, professional accreditation, and remuneration [43]. This has been done by several countries, such as India and Brazil, which have implemented programs that formally recruit and pay CHWVs, and these programs have shown a positive impact on improving community health status [44–47]. However, after years of implementation, there have been several demonstrations against the fairness of incentives given the high workload [48,49]. There were some discussions about the need for CHWVs to be a part of the civil service, but these risks relocated them from their original areas, as civil servants were required to be available in all parts of the country [49]. Another study argued that although professional CHWVs exist, there remains a need for volunteers with a limited scope of work [50]. In addition, technological improvements should be implemented to increase knowledge of CHWVs [51].

When developing CBS, it is important to optimize the use of digital technology for recording and reporting by community members, especially in the form of mobile applications, and in the monitoring and evaluation of health workers. This study found that a lack of literacy and familiarity with mobile device applications led to existing applications not being used optimally. This finding aligns with those of previous studies highlighting several challenges, such as varying literacy levels among CHVs and community members, inadequate training on digital use, and the need to use various mobile applications for different health programs [52,53]. In addition, significant challenges in the community include the cost of the device and Internet connection required to enable them to use mobile applications [54]. This is further hindered by the lack of interoperability between different disease-reporting systems, which overwhelms the number of health workers who monitor and evaluate community involvement in surveillance. Health staff must often synchronize the data entered into each application manually to ensure that the reported indicators do not differ between systems. These challenges require ensuring interoperability between systems and integration with broader surveillance and health service data collection systems [55,56].

Although the use of digital tools in CBS is becoming increasingly important, paper-based reporting may still have its place in certain contexts [17,19]. In many LMICs, CHVs have low digital literacy and educational backgrounds, making it more difficult to use electronic platforms for recording and reporting compared with health staff. Additionally, mobile applications are frequently developed by researchers and health authorities, with insufficient consideration for community needs [57]. Future development of recording and reporting systems, including mobile and web-based applications, should adopt a people-centered approach to align with the expectations and capabilities of community members.

Previous studies have demonstrated the importance of clear technical guidance and systems to ensure compatibility with wider surveillance systems in developing CBS [18,19]. Indonesia has already released technical guidance on CBS; however, limited information is available on CBS integration into wider districts, provinces, and national surveillance systems. Assessment of the integration should be conducted; for example, to what extent the health facility and authority staff can access the data collected by the community and analyse it for their work purposes. In addition to monitoring the validity and data quality, it is critical to assess the simplicity, acceptability, and flexibility of community reporting and recording systems [58]. In Indonesia, it is the MoH that designs the majority of mobile and web-based recording and reporting systems for health.

Identifying effective methods for utilizing digital data sources through hybrid systems that combine paper- and electronic-based recording and reporting by community members is critical. Hybrid systems integrating diverse data types can enhance the timeliness, accuracy, and depth of surveillance indicators, provided they undergo continuous validation [59]. A significant challenge is equipping public health experts with skills in computer science, big data, and machine learning to harness digital data sources and foster innovation while mitigating risks [60,61]. Furthermore, policies should articulate clear responsibilities for communities, emphasizing their active involvement in the early detection and reporting of cases.

This study has some limitations. First, this survey was conducted among mothers and caregivers who attended community-based health programs; they may have had better health literacy than non-attendees. Thus, the proportion of communities that participated in reporting infectious diseases may have been overestimated. Second, although purposive sampling was applied, some key respondents with unusual experience in conducting CBS were excluded from the interviews. We anticipated this by conducting triangulation meetings involving not only the informants but also wider key stakeholders to capture more varied challenges in involving the community in disease surveillance. Finally, this survey did not include questions about respondents’ experiences and challenges in the CBS for VPDs, as such systems have not been introduced. Therefore, this study may not provide a complete picture of the barriers to effective community involvement in communicable disease surveillance.

## Conclusion

This study highlights the critical role of CBS in enhancing communicable disease detection and control while identifying significant challenges and opportunities that are relevant for future development in LMICs. Strengthening CBS requires a people-centered approach to system design, focusing on community needs and capabilities. The integration of digital tools, particularly mobile applications, is essential but must address the issues of digital literacy, cost barriers, and system interoperability. Community involvement can be expanded by combining paper-based and electronic reporting to improve timeliness, accuracy, and inclusiveness, while ensuring continuous system validation. Leadership by local health authorities is vital for supervising CBS operations, validating data, and fostering community engagement. Clear policies that define community roles and emphasize education, communication, and active participation are required. Finally, equipping public health professionals with skills in data science and digital innovation will drive the advancement of CBS, ensuring its integration into resilient public health frameworks.

## Supplementary Material

Sitorukmi Rusadi_CBS VPD_Supplementary File_revision.docx

## Data Availability

The datasets generated and analysed during the current study are not publicly available due to institutional policy and participant confidentiality. Interested researchers may request access by contacting the corresponding author.
