# Supplementary material for: Developing community-based surveillance systems for vaccine-preventable diseases: lessons learned from Indonesia
Source: Glob Health Action. 2025 Aug 28;18(1):2548083. doi: 10.1080/16549716.2025.2548083 (PMC12395612; doi:10.1080/16549716.2025.2548083)
Supplement: Sitorukmi Rusadi_CBS VPD_Supplementary File_revision.docx [file ZGHA_A_2548083_SM8518.docx]

Figure A. Flow of respondent recruitment


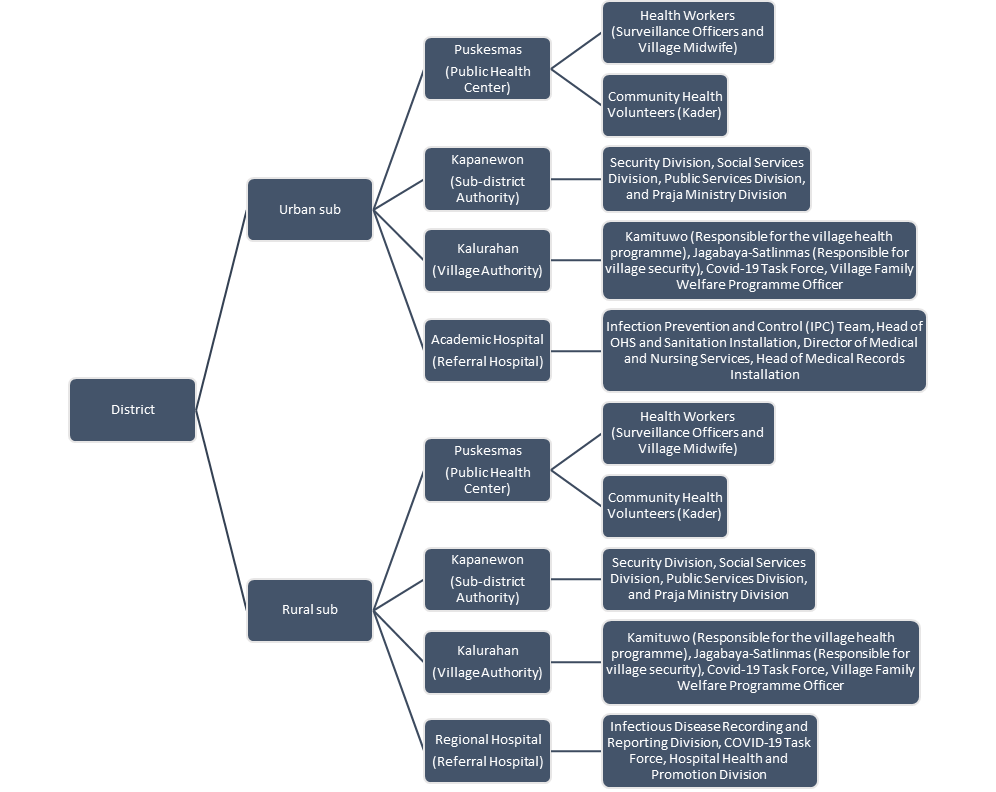


Table A. Top 5 most frequently participated community-based surveillance activities by the respondents

| Type of surveillance activities | Yes | | No | | Not knowing the cases | |
| --- | --- | --- | --- | --- | --- | --- |
|  | **n** | **%** | **n** | **%** | **n** | **%** |
| Assistance in disseminating COVID -19 information | 31 | 44.3% | 39 | 55.7% | 0 | 0.0% |
| Participation in COVID -19 data collection activities | 20 | 28.6% | 50 | 71.4% | 0 | 0.0% |
| Involvement in community groups for sharing COVID-19 information | 8 | 11.4% | 62 | 88.6% | 0 | 0.0% |
| Reporting cases of disease via SMS or WhatsApp | 21 | 30.0% | 49 | 70.0% | 0 | 0.0% |
| Participation in other infectious disease data collection activities | 5 | 7.1% | 63 | 90.0% | 2 | 2.9% |

Table B. Entities that are recommended to be involved in community-based surveillance

| Entities involved in community-based surveillance | N | Percentage |
| --- | --- | --- |
| Neighbourhood committee (RT) | 43 | 61.4% |
| Community health worker (*kader*) | 41 | 58.6% |
| Hamlet committee (RW) | 29 | 41.4% |
| Housewife | 14 | 20.0% |
| Head of Household | 14 | 20.0% |
| Subdistrict Head | 11 | 15.7% |
| Family Welfare Movement (*Dasawisma*) | 5 | 7.1% |
| Hospital Staff | 4 | 5.7% |
| Others | 41 | 58.6% |

**Others: Midwife, hamlet leader, entire community, oneself, community figure, village head, community facilitator, youth*

Figure B. Willingness to participate in community-based surveillance by age group

Table C. Types of community-based surveillance activities according to respondents' perceptions

| Type Community-Based Surveillance Activities | Frequency (N=43) | Percentage |
| --- | --- | --- |
| Case reporting | 9 | 20.9% |
| Program socialization | 9 | 20.9% |
| Case data collection/survey | 7 | 16.3% |
| Awareness-raising | 5 | 11.6% |
| Support environmental health* | 5 | 11.6% |
| Assisting the sick** | 3 | 7.0% |
| Any activities | 3 | 7.0% |
| Willingness to be recorded | 2 | 4.7% |

**PHBS: Monitoring larvae, environmental cleanliness, dengue prevention, disease prevention*

***Accompanying and visiting sick individuals, and participating in integrated health posts (posyandu)*
